# Report of Parasitic Entozoa Encountered in General Practice in Texas during over Forty Years

**Published:** 1894-06

**Authors:** F. Herff

**Affiliations:** San Antonio, Texas


### TEXAS MEDICAL JOURNAL.

ESTABLISHED JULY, 1885.

PUBLISHED MONTHLY.—SUBSCRIPTION $2.00 A YEAR.

VOL. IX. AUSTIN, JUNE, 1894. NO. 12.

### Original Contributions.

For Texas Medical Journal.

### REPORT OF PARASITIC ENTOZOA ENCOUNTERED IN GENERAL PRACTICE IN TEXAS DURING OVER FORTY YEARS.

BY F. HERFF, M. D., SAM ANTONIO, TEXAS.

[Read before the Texas State Medical Association at Austin, April 26, 1894.]

IN ANSWER to a request from the chairman of the Section on Microscopy and Pathology that Dr. Herff should place on record before the Section the results of his very wide opportunities for observation in relation to the parasites encountered in his long and honorable career in medicine in the western portion of this State, Dr. Herff replied as follows:

It is with bad conscience that I answer your kind letter written to me several weeks ago. From its contents, as well as from the courteous and polite manner you address me, it ought to have been answered immediately. I tender you my sincere apologies, and have no other excuse to offer for my remissness than my bad health, and a crippled hand (caused by a wound during an operation), which made writing very difficult for me. I hardly think I can go to Austin; but if you have any use for my helminthological cases, I will send you a short abstract of them in this letter. You may use it as you think proper, and if not acceptable, throw it into the waste-basket.

During my practice in San Antonio I have observed the following intestinal and muscular entozoa:

1. *Tænia mediocanellata* is the most common of all, so much so that in some localities it occurs in nearly every family. Strange to say, I have never encountered the cysticercus of this species in beef or veal, nor yet in man. Perhaps I did not devote enough time to find it, but it must undoubtedly be very rare or very much scattered over the infected animals. Otherwise I would certainly have met with it, as I did very often with the cysticercus of the tænia solium in the pig.

2. *Tænia solium* is also very common, principally in the German settlements. The cysticercus is very often met in the pork. I will remark that I have frequently met with living and moving scolices of tænia in well-water, and am confident they were introduced into the wells by contamination from privies or by carelessly cleaning chamber vessels near the well. Wherever such scolices were found, one or several members of the family had tænia, which in some instances was discovered in consequence of the finding of the scolices in the well.

3. *Bothriocephalus latus* I have found only about twelve times, and do not believe that the animal is indigenous to Texas. The majority of the specimens found occurred in Poles and Swiss who had undoubtedly imported the parasite. I believe, however, that it must be indigenous in Mexico, as I have seen two cases in persons coming from the other side of the Rio Grande.

4. *Tænia echinococcus* is a common parasite among the dogs of this district; I have found it in all the dogs I have dissected for one or another reason. It is a very common parasite in the muscles and liver of the jack-rabbit, so much so that many persons abstain from eating its meat altogether. It is also not uncommon in the human body, a curious fact in comparison with the absence of the cysticercus. I have found it three times in the liver, once in the extensor muscles of the thigh (or rather between them), once in the kidney and bladder, and once in the bulb of the eye. All these cases were verified by the discovery of the hooklets. One case of liver echinococcus healed spontaneously, after vomiting and evacuating per anum a great many cysts; two cases were operated upon, of whom one died because of the carelessness of the nurse, who administered, by mistake, pure carbolic acid instead of castor oil, ten days after the operation. The case of echinococcus in the leg recovered. The amount of cysts in all these cases was very great, at least from one to two quarts of cysts being found in each case. The echinococcus of the bulb was discovered by accident, in an eye which I enucleated for panophthalmitis threatening the other eye. The parasite filled the bulbus, and was easily recognized by the hooklets. I do not know that echinococcus has, before this, been described as occurring in the eye; while cysticercus is not very uncommon in the eye in some parts of Germany.

5. *Trichina spiralis* I have found in eighteen persons belonging to five families. In all cases the infected meat was examined and trichinæ found. In one case, I extracted muscular trichinæ with the harpoon of Duchesne, from the forearm of a girl. In another instance the infected animal was a bear, killed near Bandera, by some hunters who had trichinosis afterwards. Here also the meat was examined, and the trichinæ found. Most remarkably, there was no death in all the eighteen cases, although some of them came very near dying. A few cases were very light, and presented only slight facial œdema and muscular pains. If one considers the great fatality of trichinosis in Europe, the demand for a reason for the difference naturally occurs; I believe it is to be explained by the condition of the encapsulated trichniæ of the pork. In Europe, hogs are very often allowed to reach an age of two or three years before they are killed; moreover, they are nearly always stall-fed, under which circumstances the opportunity to acquire the trichinæ from the rat is given at an early age. Consequently, the trichinæ are nearly always surrounded by a calcareous shell, and are nearly indestructible. In Texas, the pigs run wild during the first six months, and can only acquire trichinæ during the few weeks they are fed in the pig-sty. At the age of ten months, the most of them are butchered; consequently their trichinæ, should they have any, are not surrounded as yet by as resistant or as calcareous shells as in the former case, and are much more easily destroyed by salting or cooking. In fact, among all the trichinæ I have found in pork in Texas, I have never yet met one with a calcified shell.

6. *Ascaris lumbricoides* is seen very frequently, but by no means as commonly as in other countries; it scarcely ever causes any disturbances of any consequence. It is said to be quite common among the negroes, but this I am not able to confirm from personal observation.

7. *Trichocephalus dispar* I have never met with in Texas, probably because the parasite never causes much trouble.

8. *Anchylostomum duodenale* I believe I have found at a postmortem examination of the body of a Mexican lady who died from anæmia and chronic dysentery. She had been sick for several years, and always insisted that she had occasionally small worms, a statement which was disbelieved, as no one but she had seen them. In her duodenum, embedded in bloody mucus, I found a large number of worms, not much longer than three-fourths of an inch, which I then believed (thirty year ago), in my ignorance, to be young ascarides. I have encountered several cases since then, in Italians, presenting the same train of symptoms; but none of these cases died, and it was impossible to verify the diagnosis of anchylostoma, as the worm is hardly ever met in the stools. I believe, however, that these were instances of parasitism hy anchylostoma, and I feel convinced on reflection that the worms found in the duodenum of the first case were really specimens of this entozoon.

9. I have met with a peculiar form of hæmaturia without renal or vesical pain, with great weakness and cachexia, and with slight œdema of the face, and I have thought that perhaps these cases were due to the presence of the *Bilharzia hæmatobium* (*distomum Bilharzii*). Undoubtedly, the cases were neither chronic Bright's disease nor acute nephritis, nor malarial hæmaturia, and all recovered after six or eight weeks' illness. I examined the urine for the eggs of the distomum, but never found any, which, however, might be accounted for by my want of experience with the parasite. It is said the disease is not uncommon on the Rio Grande, and is treated successfully there by the administration of an infusion of *oci de lé*, an herb which properly belongs to the fanily of the *compositæ*. I have procured it from Mexico, and used it successfully in my cases.

10. I have several times met with cases of *elephantiasis scroti*, *clitoridis*, and *femoris*, and as that affection seems to be connected with the existence in the body of the *filaria sanguinis hominis*, I suspect that that parasite is also to be met with in Texas. I have never, however, recognized the entozoon, nor have I ever seen a case of chyluria, which is said to be caused by it.

